# Supplementary material for: Wnt5a and Notum influence the temporal dynamics of cartilaginous mesenchymal condensations in developing trachea
Source: Front Cell Dev Biol. 2025 Apr 9;13:1523833. doi: 10.3389/fcell.2025.1523833 (PMC12015613; doi:10.3389/fcell.2025.1523833)
Supplement: Supplementary file 2 [file Table1.pdf]

Supplementary Table 1

Wnt5af/f	F: GGT GAG GGA CTG GAA GTT GC<br>R: GGA GCA GAT GTT TAT TGC CTT C
Dermo1Cre	F: TGC CAC GAC CAA GTG ACA GCA ATG<br>R: AGA GAC GGA AAT CCA TCG CTC G
Notum	F: CTG ACA GCA TGC TCT GTG CG<br>R: ACT ATT CTG CAG ACC GAG CCA GTC
Sox9KIeGFP	1: GAG GGG CTT GTC TCC AG<br>2: ACA CCG GCC TTA TTC CAA G<br>3: GGC AGC TAC TCT TGA AAT CCA
Gamma SMA	F: CCT ACG GCG TGC AGT GCT TCA GC<br>R: CGG CGA GCT GCA CGC TGC GTC CTC
