# Supplementary material for: Wnt5a and Notum influence the temporal dynamics of cartilaginous mesenchymal condensations in developing trachea
Source: Front Cell Dev Biol. 2025 Apr 9;13:1523833. doi: 10.3389/fcell.2025.1523833 (PMC12015613; doi:10.3389/fcell.2025.1523833)
Supplement: Supplementary file 3 [file Table2.pdf]

PRIMARY ANTIBODY	REFERENCES	SPECIES	DILUTION	COMPANY	CATALOG #
Anti Actin, Alpha Smooth Muscle-Cy3, monoclonal	[1]	Mouse	1:200	Sigma Aldrich	C6198
Anti Actin, Alpha Smooth Muscle, monoclonal	[2]	Mouse	1:200	Sigma Aldrich	A5228
Anti-Sox9, polyclonal	[2]	Rabbit	1:200	Millipore	AB5535
Human Sox9, polyclonal	[3]	Goat	1:50	R&D	AF3075
Nkx2.1	[4]	Rabbit	1:200	Seven Hills Bioreagents	R1231
Nkx2.1	This study	Guinea Pig	1:200	Seven Hills Bioreagents	GP237
Anti-Mo CD326 (EpCAM) APC	[5]	Mouse	1:200	Invitrogen	17-5791-82
Anti-N Cadherin	[6]	Rabbit	1:100	Abcam	Ab76057
**SECONDARY ANTIBODY**					
Donkey anti Mouse IgG (H+L), 350			1:200	Invitrogen	A10035
Donkey anti Rabbit IgG (H+L), 350			1:200	Invitrogen	A10039
Donkey anti Goat IgG (H+L), 488			1:200	Jackson Immuno	705-546-147
Donkey anti Guinea Pig (H+L), 488			1:200	Jackson Immuno	706-545-148
Donkey anti Mouse IgG (H+L), 488			1:200	Invitrogen	A21202
Donkey anti Rabbit IgG (H+L), 488			1:200	Invitrogen	A21206
Donkey anti Mouse IgG (H+L), 594			1:200	Invitrogen	A21203
Donkey anti Rabbit IgG (H+L), 594			1:200	Invitrogen	A21207

1. Yin, W., et al., *The potassium channel KCNJ13 is essential for smooth muscle cytoskeletal organization during mouse tracheal tubulogenesis.* Nat Commun, 2018. **9**(1): p. 2815.
2. Snowball, J., et al., *Endodermal Wnt signaling is required for tracheal cartilage formation.* Developmental biology, 2015. **405**(1): p. 56-70.
3. Gerhardt, B., et al., *Notum attenuates Wnt/betacatenin signaling to promote tracheal cartilage patterning.* Developmental biology, 2018.

4. Cornett, B., et al., *Wntless is required for peripheral lung differentiation and pulmonary vascular development.* Developmental biology, 2013. **379**(1): p. 38-52.
5. Kuwahara, A., et al., *Delineating the early transcriptional specification of the mammalian trachea and esophagus.* Elife, 2020. **9**.
6. Piprek, R.P., et al., *N-Cadherin Is Critical for the Survival of Germ Cells, the Formation of Steroidogenic Cells, and the Architecture of Developing Mouse Gonads.* Cells, 2019. **8**(12).
