# Supplementary figures and images for: Wnt5a and Notum influence the temporal dynamics of cartilaginous mesenchymal condensations in developing trachea
Source: Front Cell Dev Biol. 2025 Apr 9;13:1523833. doi: 10.3389/fcell.2025.1523833 (PMC12015613; doi:10.3389/fcell.2025.1523833)

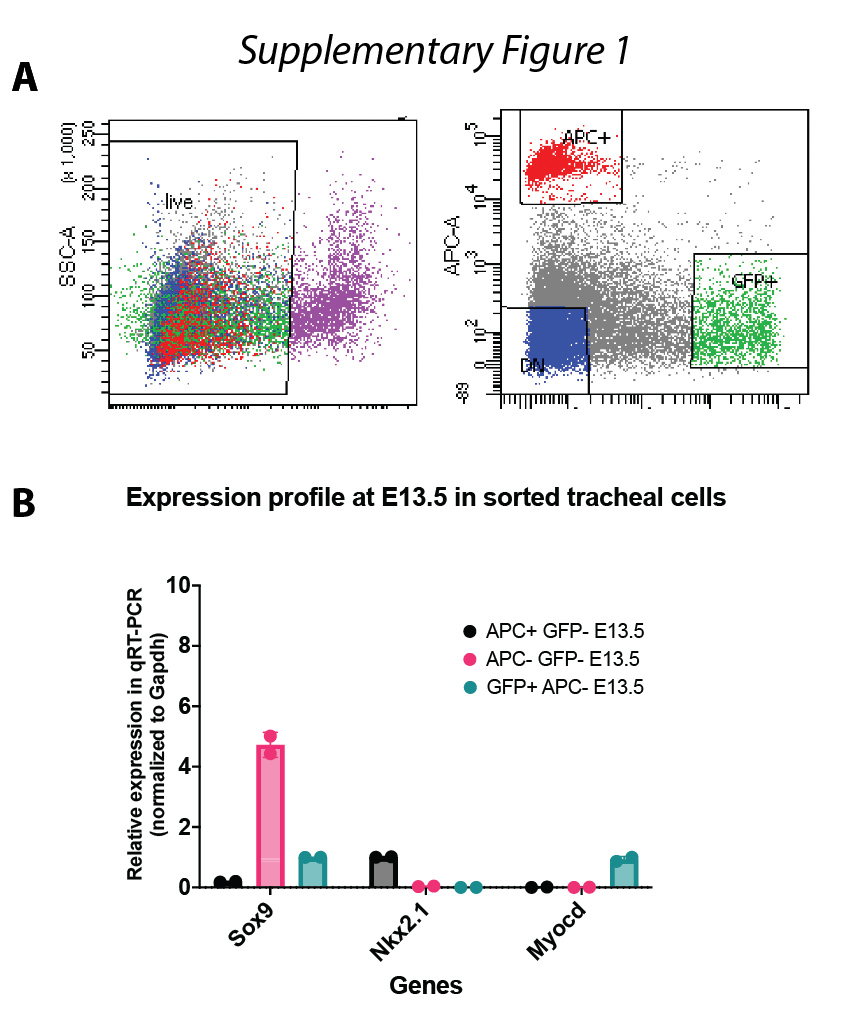

Supplement: Supplementary file 4 [file Image1.jpeg]

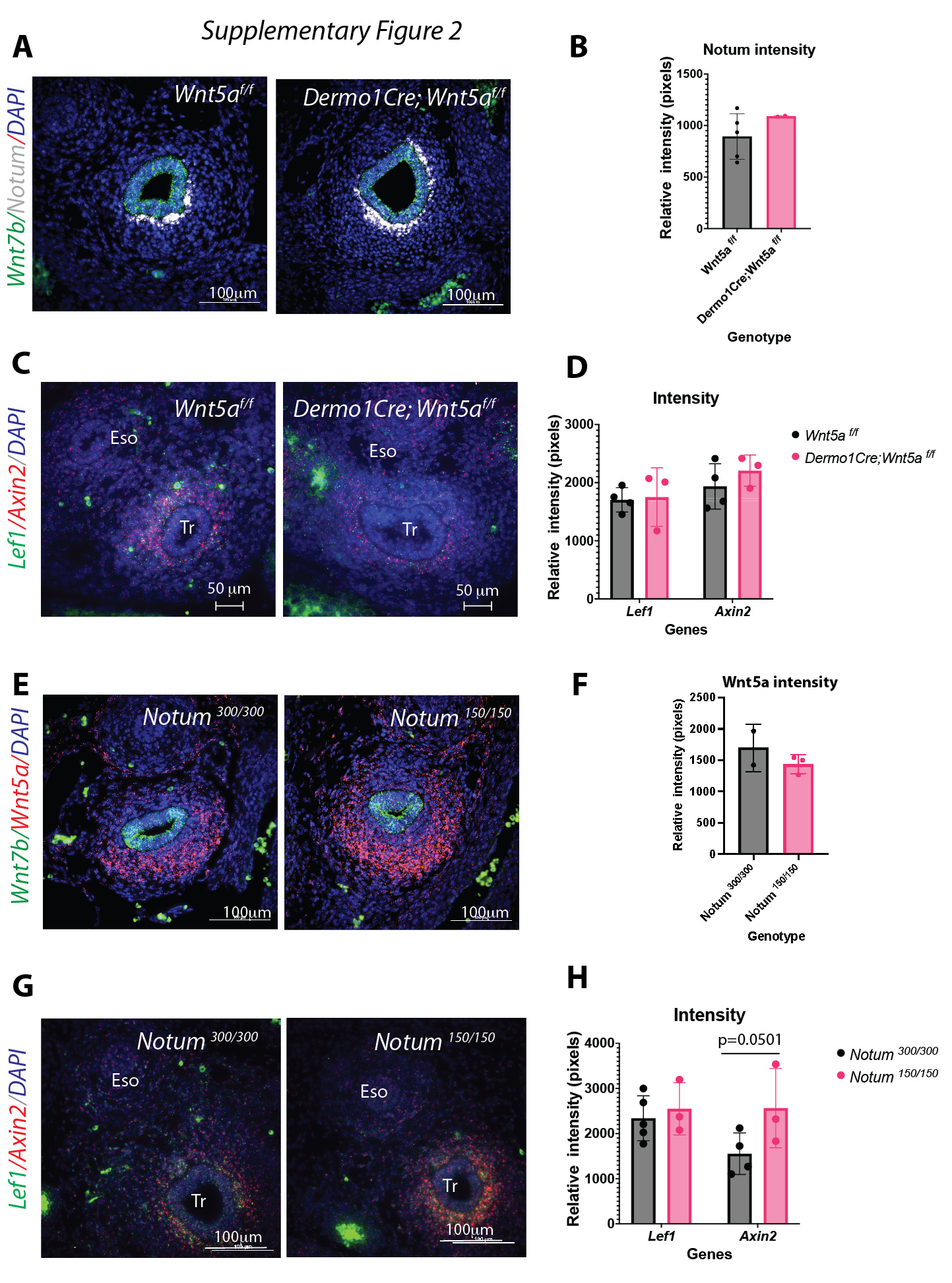

Supplement: Supplementary file 5 [file Image2.jpeg]
