# Supplementary material for: Wnt5a and Notum influence the temporal dynamics of cartilaginous mesenchymal condensations in developing trachea
Source: Front Cell Dev Biol. 2025 Apr 9;13:1523833. doi: 10.3389/fcell.2025.1523833 (PMC12015613; doi:10.3389/fcell.2025.1523833)
Supplement: Supplementary file 6 [file DataSheet1.pdf]

SUPPLEMENTARY MATERIAL

Table1:

Primers utilized for genotyping.

Table 2:

Antibodies used for the studies.

Supplementary Figure1: Cell sorting strategy distinguishes major cell types in developing trachea.

A) Dot plots and density plots display the gating strategy utilized to isolate epithelial (APC+), muscle cells (GFP+), and chondroblasts (APC-GFP-, double negative DN) from the total pool of live cells. B) Gene expression profile as determined by qRT-PCR confirmed the identity of the different cell populations. *Sox9* is highly expressed by APC-GFP- cells. *Myocd* transcripts are present in GFP+ cells but not detected in APC+ or APC-GFP- cells. *Nkx2.1* expression was only detected in APC+ cells.

Supplementary Figure2: Regulation of Wnt targets after deletion of Wnt5a and Notum

A) RNA scope in situ hybridization demonstrates that levels of levels of Notum are not affected after deletion of *Wnt5a*, based on staining and mean intensity quantification (B) (N=3-4). C) Likewise, levels of *Lef1* and *Axin2* transcripts are not increased after deletion of Wnt5a. D) Mean intensity quantification for panel C. E) *Wnt5a* transcript levels are not affected after deletion of *Notum*. F) Mean intensity quantification for panel E. G) RNA in situ hybridization after *Notum* deletion shows increased levels of transcripts for *Axin2*. H) Mean intensity levels for panel G, N=3-4. Representative images are shown. Eso= esophagus, Tr= trachea.

Video:

Time-lapse imaging depicts the cartilaginous mesenchymal condensation in *Sox9KI eGFP* trachea lung tissue subjected to different conditions. Tissue was isolated at E12.5 and cultured at the air-liquid interphase. Timelapse videos were recorded overnight, 10x

images were acquired every 30 minutes, between 24 and 42 hours from the beginning of the incubation. In vehicle treated (DMSO) samples, condensations were observed around 30 hours post incubation (video 1), while Treatment with ABC99, a Notum inhibitor, prevented cartilaginous mesenchymal condensations at 42 hours compared to control (arrow in the video 2). On the other hand, KN93 (calmodulin kinase (CamK) inhibitor) and the JNK inhibitor treatments cause earlier condensation at 24 hours of incubation compared to controls (arrows in videos 3 and 4). Note that neither treatment caused toxicity as pulmonary branching occurred uneventfully (asterisk in the lung) regardless of the chemical addition.
